# Anti-VEGF for the Management of Diabetic Macular Edema

**DOI:** 10.1155/2014/632307

**Published:** 2014-02-05

**Authors:** Francisco Rosa Stefanini, Emmerson Badaró, Paulo Falabella, Michael Koss, Michel Eid Farah, Maurício Maia

**Affiliations:** ^1^Retina Division, Retina Division, Department of Ophthalmology and Visual Sciences, Federal University of São Paulo (UNIFESP), 821 Botucatu Street, 2nd Floor, 04023-062 São Paulo, SP, Brazil; ^2^Doheny Eye Institute, Department of Ophthalmology, Keck School of Medicine, University of Southern California, 1450 San Pablo Street, Los Angeles, CA 900033, USA; ^3^Department of Ophthalmology, Goethe University, 7 Theodor Stern Kai, 60590 Frankfurt am Main, Germany

## Abstract

Diabetic retinopathy (DR) is an important cause of vision loss around the world, being the leading cause in the population between 20 and 60 years old. Among patients with DR, diabetic macular edema (DME) is the most frequent cause of vision impairment and represents a significant public health issue. Macular photocoagulation has been the standard treatment for this condition reducing the risk of moderate visual loss by approximately 50%. The role of vascular endothelial growth factor (VEGF) in DR and DME pathogenesis has been demonstrated in recent studies. This review addresses and summarizes data from the clinical trials that investigated anti-VEGF for the management of DME and evaluates their impact on clinical practice. The literature searches were conducted between August and October 2013 in PubMed and Cochrane Library with no date restrictions and went through the most relevant studies on *pegaptanib*, *ranibizumab*, *bevacizumab*, and *aflibercept* for the management of DME. The efficacy and safety of intravitreal anti-VEGF as therapy for DME have recently been proved by various clinical trials providing significantly positive visual and anatomical results. Regarding clinical practice, those outcomes have placed intravitreal injection of anti-VEGF as an option that must be considered for the treatment of DME.

## 1. Introduction

**Figure 1:**
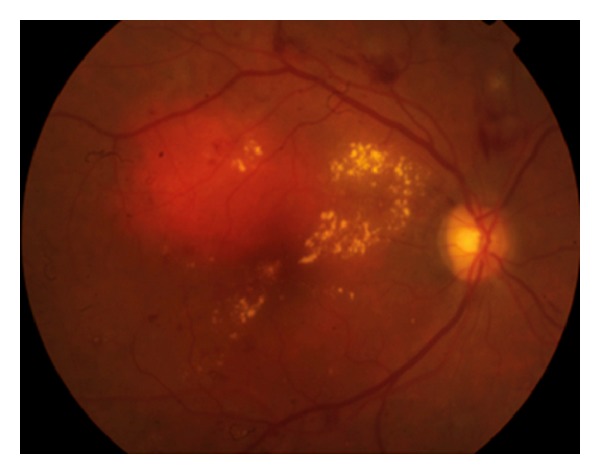
Diabetic retinopathy showing intraretinal hemorrhages, hard exudates, and microaneurysms in the posterior pole associated with diabetic macular edema.

**Figure 2:**
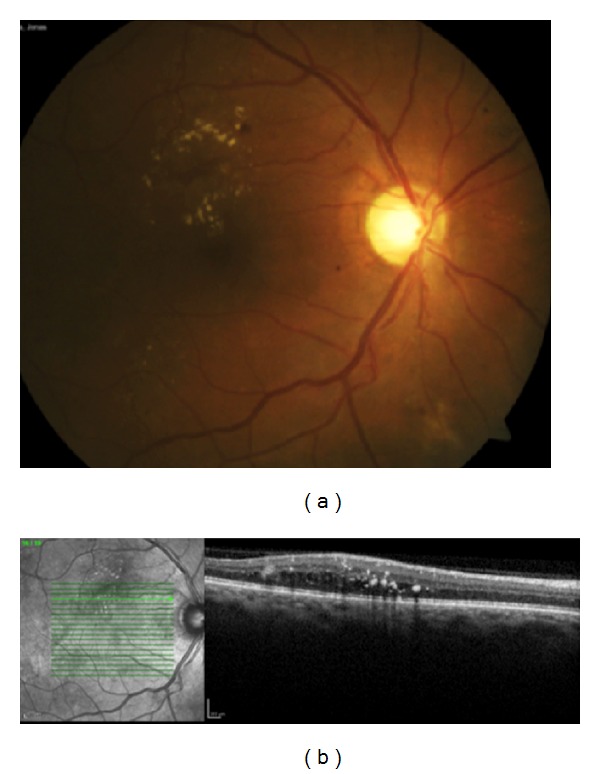
(a) Fundus photograph of the right eye of a patient with diabetic retinopathy with hard exudates and focal edema temporal superior to the macula. (b) Optical coherence tomography of the patient showing intraretinal edema and hard exudates.

Obesity is a major risk factor for type 2 diabetes and has increased in prevalence in the last decades [1, 2]. Diabetic retinopathy (DR) is a leading cause of vision loss in working-age patients around the world. One percent of all cases of blindness worldwide can be attributed to DR [3, 4]. Diabetic macular edema (DME) is primarily responsible for vision impairment in diabetic patients [5–7] (Figure 1). A large epidemiological study indicated that 26% of patients with diabetic retinopathy presented with DME [8]. According to another study, the prevalence of macular edema in patients with recently diagnosed diabetes is 0 to 3%, increasing to 29% in diabetic patients with over 20 years of disease [9]. Therefore, ophthalmic complications of the diabetes, especially DME, represent a significant public health issue (Figure 2).

Both proliferative and nonproliferative DR may show DME, which is classified as either focal, if edema is caused by a focal leakage from microaneurysms, or diffuse, if generalized leakage from retinal capillaries with abnormal permeability is observed throughout the posterior pole [10–12]. Besides the abnormal permeability, edema may also occur due to occlusion of the capillary bed that leads to dilation of the patent capillaries and leakage [13].

Controlling DME risk factors such as systemic hypertension, hyperlipidemia, and poor blood glucose control may decrease the development of edema and lower progression of DR [14]. Other risk factors are adult-onset diabetes mellitus, cardiovascular disease, impaired renal function, advanced DR, increased number of retinal microaneurysms, and vitreomacular traction [13, 15].

The Early Treatment Diabetic Retinopathy Study (ETDRS) showed the benefit of focal/grid laser for the management of DME, reducing the risk of moderate visual loss by approximately 50%, and since then, macular photocoagulation (MPC) has been the gold standard treatment [16]. Recently, data from the Diabetic Retinopathy Clinical Research Network (DRCR.net) studies demonstrated best-corrected visual acuity (BCVA) improvement of more than 5 letters of vision in 51, 47, and 62% of eyes treated with monthly 0.5mg of intravitreal ranibizumab after 1, 2, and 3 years of follow-up, respectively [7, 17–19].

**Figure 3:**
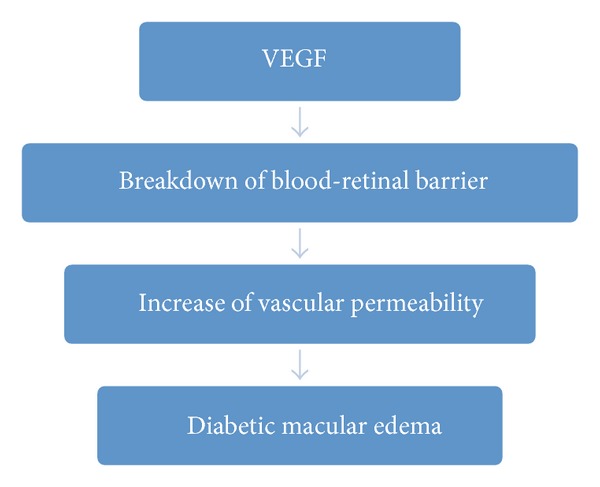
VEGF and pathophysiology of diabetic macular edema.

Vascular endothelial growth factor (VEGF) is an important mediator of blood-retinal barrier breakdown, which leads to fluid leakage and the development of macular edema (Figure 3) [20]. Observing that VEGF intraocular levels are increased in DME, it was hypothesized that alternative or adjunct therapies using VEGF inhibitors (anti-VEGF) could be beneficial in reversing vision loss from macular edema [21].

The aim of this review was to address and compare, where possible, data from the clinical trials that assessed anti-VEGF for the management of DME and to evaluate their impact on clinical practice.

## 2. Methods

The literature searches were conducted between August and October 2013 in PubMed and Cochrane Library with no date restrictions. Relevant unpublished data regarding the topic “anti-VEGF for the management of diabetic macular edema” presented at official retina conferences during this period were also considered in this review. The search strategy used the following words: *diabetic retinopathy*, *diabetic macular edema*, *vascular endothelial growth factor*, *anti-VEGF*, *pegaptanib*, *Macugen*, *bevacizumab*, *Avastin*, *ranibizumab*, *Lucentis*, *aflibercept*, *VEGF Trap Eye*, and *Eylea*.

## 3. Results and Discussion

On the basis of evidence that VEGF expression and signaling are deregulated in diabetic retinopathy, anti-VEGF compounds have been studied as a pharmacological alternative treatment for DME. Considering agents originally used to treat neovascular age-related macular disease (AMD), recent trials have addressed the efficacy and safety of different types of anti-VEGF in the treatment of DME, including pegaptanib (Macugen, OSI/Eyetech, USA), ranibizumab (Lucentis, Genentech, Inc., USA), bevacizumab (Avastin, Genentech, Inc., USA), and aflibercept (EYLEA, Regeneron Pharmaceuticals, Inc., USA). 

Pegaptanib sodium is a selective VEGF antagonist that binds with the 165 isoform of VEGF and was approved by the US Food and Drug Administration (FDA) for the treatment of neovascular AMD [22]. Ranibizumab is a recombinant humanized immunoglobulin G1 kappa antibody fragment that binds with and inhibits the biologic activity of all isoforms of human VEGF-A. It was approved by the FDA for the treatment of neovascular AMD, macular edema associated with retinal vein occlusion, and since 2012, it is approved for the treatment of DME [23]. Bevacizumab is a full-size, humanized, recombinant monoclonal IgG antibody that inactivates all VEGF-A isoforms and is approved for systemic use in the treatment of certain metastatic cancers, but its use for ocular diseases is off-label. Aflibercept, or VEGF Trap-Eye, is a new, fully human, 115 kDa recombinant fusion protein that binds with and inhibits all isoforms of 4 VEGF-A and B as well as binds placental growth factors 1 and 2. It has the advantages of a longer half-life in the eye and a higher binding affinity for VEGF-A [24]. VEGF Trap-Eye was approved for the treatment of AMD in 2011 (Table 1) [25].

### 3.1. Pegaptanib

A phase II randomized double-masked multicenter controlled trial investigated different doses of intravitreal pegaptanib (0.3, 1, and 3 mg) and sham injections in patients with diabetic macular edema. Data published in 2005 showed that 172 individuals with DME involving the center of the macula were included with BCVA at baseline between 20/50 and 20/320. Injections were given at study entry, week 6, and week 12. Additional injections and/or laser therapy could be performed as needed, after week 12 until the end of week 36. Subjects receiving pegaptanib had better BCVA outcomes compared to sham at week 36, with a larger proportion of those receiving 0.3 mg of the drug having a visual acuity gain of 2 lines or more (34 versus 10% *P* = 0.003). The same positive results in favor of 0.3 mg pegaptanib were observed with regard to reduction of central retinal thickness. Subjects assigned to pegaptanib were less likely to need additional laser therapy [26].

In 2011, a phase-2/3, multicenter, randomized, double-blinded trial conducted in the United States included 260 subjects with DME involving the center of the macula and BCVA at baseline between 20/50 and 20/200. They received 0.3 mg of either intravitreal pegaptanib or sham injection every 6 weeks and were followed for 102 weeks. At week 18, macular grid/focal laser was performed as needed, based on ETDRS criteria. The primary efficacy endpoint was the proportion gain of 10 letters or more of visual acuity (VA) from baseline to year 1. No safety issues were identified throughout the study. Again pegaptanib was superior to sham injection regarding visual acuity gain at the end of the first year (37 versus 20%; *P* = 0.0047). The group treated with pegaptanib gained 6.1 letters in mean BCVA at week 102, while the sham injection group gained 1.3 letters (*P* < 0.01). Significantly fewer macular laser indications were observed in the pegaptanib group compared to sham injection [27].

### 3.2. Ranibizumab

A small pilot study, in 2006, provided early data proving that intravitreal ranibizumab was effective and improved vision acuity in patients with DME. Ten diabetic patients with chronic macular edema were included and received intravitreal ranibizumab (IVR) at study entry and at months 1, 2, 4, and 6. At month 7, the study showed an improvement in mean visual acuity and reduction in mean foveal thickness, demonstrating the importance of VEGF in the pathophysiology of DME [28].

A multicenter, phase II trial, READ-2, was conducted randomizing 126 subjects with DME evenly into 3 groups: group 1 received 0.5 mg of ranibizumab at baseline and months 1, 3, and 5; group 2 received focal/grid laser at baseline and at month 3 if needed; and group 3 received a combination of focal/grid laser and 0.5 mg ranibizumab at baseline and at month 3. The primary outcome was mean change in BCVA at 6 months. Group 1 (+7.24 letters) was superior to group 2 (−0.43 letters, *P* = 0.01) regarding ETDRS BCVA, while improvement in group 3 (+3.80 letters) was not significant compared to the other two groups. A visual gain of 3 lines or more was observed in 22% in group 1, 0% in group 2, and 8% in group 3 (*P* = 0.002) [29].

Another phase II clinical trial, RESOLVE, randomized 151 patients with DME to receive either 0.3 or 0.5 mg of ranibizumab as monotherapy versus sham injection, monthly for 3 months. After one month, patients were allowed to have their doses doubled to 0.6 mg or 1 mg (or double sham) if indicated by specific study criteria. Both groups were eligible for rescue laser on the basis of foveal thickness and visual acuity. Patients in the sham group had their doses doubled more often (91.8 versus 68.6%), as well as rescue laser being more often performed in the sham group (34.7 versus 4.9%). The ranibizumab group had BCVA improvement averaging +10.3 letters at 1 year, while the sham group had +1.4 letters (*P* < 0.001); the same superiority was observed in central retinal thickness improvement, −194.2 versus −48.4 *μ*m in the ranibizumab and sham groups, respectively (*P* < 0.001). Visual gain of 10 letters or more was observed in 60.8% of the ranibizumab-treated patients, compared with 18.4% of the sham-treated patients (*P* < 0.001) [30].

The RESTORE phase III clinical trial conducted in Europe randomized 345 subjects into 3 different groups: (A) receiving 0.5 mg of ranibizumab and sham laser, (B) receiving 0.5 mg of ranibizumab and active laser, and (C) receiving laser and sham injection. Monthly treatment was given for 3 months followed by “as needed” treatment. In the 12-month report, visual acuity improvement was 6.1 letters in group A, 5.9 letters in group B, and 0.8 letters with laser alone in group C. There was a statistically significant difference between both ranibizumab groups and the laser group (*P* < 0.0001), but no differences were seen between the ranibizumab groups. Mean central retinal thickness also decreased significantly in both ranibizumab groups compared with laser alone. The mean number of injections was 7 in the ranibizumab group A and 6.8 in the ranibizumab plus laser group B. No safety issue was observed in this study [31].

Two methodologically identical phase III trials, RIDE and RISE, were intended to support FDA approval of ranibizumab for treatment of DME and were sponsored by Genentech (Genentech Inc., South San Francisco, USA). The parallel, multicenter, double-masked, sham injection-controlled, randomized studies were conducted in the United States and South America. RIDE enrolled 377 patients with DME and RISE enrolled 382. They were evenly assigned to 3 different groups to receive either 0.3 or 0.5 mg of ranibizumab or to receive sham injections, monthly treatment for 24 months. At 3 months, rescue laser was allowed for all patients. After 24 months, the protocol was changed and all patients previously assigned to sham injections became eligible to receive 0.5 mg ranibizumab injection [32].

At 2 years, RISE and RIDE outcomes showed significant superiority of both ranibizumab groups over the sham injection groups regarding improvement of visual acuity and reduction of central retinal thickness. The primary efficacy point was improvement of 15 letters or more, and considering the sham injection, 0.3 mg ranibizumab, and 0.5 mg ranibizumab groups, the achievement rate was 18.1, 44.8, and 39.2% of patients in RISE and 12.3, 33.6, and 33.3% of patients in RIDE. It is worth noting that in the RIDE and RISE studies there was no direct comparison between ranibizumab and laser, due to a 3-month delay in laser treatment, even in the sham groups. Similarly to other ranibizumab trials, safety findings were acceptable. Endophthalmitis occurred at a rate of 0.8%. The incidence rates of nonfatal myocardial infarction, cerebrovascular accident, and death from vascular or unknown causes were 4.9–5.5% in the sham groups and 2.2–8.8% in the ranibizumab groups. Based on these trials, FDA approved ranibizumab as the first anti-VEGF for the treatment of DME.

The same primary endpoint was evaluated at 36 months and the visual effects were maintained. Improvement of 15 letters or more, in the sham injection, 0.3 mg ranibizumab, and 0.5 mg ranibizumab groups was, respectively, 22.0, 41.6, and 51.2% in RISE patients and 19.2, 36.8, and 40.2% in RIDE patients [33].

The Diabetic Retinopathy Clinical Research Network (DRCR.net) conducted a study with a more complicated design. Although other trials had shown the benefits of anti-VEGF as a treatment for DME, monthly injections or monthly evaluations were not feasible in clinical practice. DRCR.net protocol I tried to give more flexibility to the treatment and to differentiate between the effect of ranibizumab and laser. A total of 854 patients with DME were randomized into 4 groups: sham injection plus prompt laser; 0.5 mg ranibizumab plus prompt laser, 0.5 mg ranibizumab plus deferred laser (at or after 24 weeks), and 4 mg triamcinolone plus prompt laser. Treatment was given according to the “4 : 2 : 7 rule”: four monthly injections; additional injections if required at the next 2 study visits, and 7 subsequent study visits during which injection could be indicated at the investigator's discretion if the study eye was considered to show “no improvement” [17].

The primary outcome was mean change in BCVA at 1 year and the findings showed that both ranibizumab groups, with prompt or deferred laser, gained 9 letters, superior to the triamcinolone plus laser and sham plus laser groups, which gained 4 and 3 letters, respectively. At 2 years, improvements in mean change in BCVA were maintained and fewer injections were performed in the ranibizumab groups throughout the second year: from 8 in the prompt laser and 9 in the deferred to 2 and 3, respectively. It is noteworthy that the number of injections was similar between prompt laser group and deferred laser group [17].

Data from the third year of protocol I suggest that early initiation of focal/grid laser treatment not only lacks benefit but also may be detrimental to visual outcomes, since the deferred laser group showed 57% of patients gaining 10 letters or more and 5% losing 10 letters or more, while the prompt laser group showed 42% gaining 10 or more letters and 10% losing 10 or more letters [34].

An exploratory analysis of protocol I was performed to evaluate the effect of intravitreal ranibizumab and triamcinolone on worsening diabetic retinopathy. Despite acknowledging the limitations of exploratory analysis, the results indicated that ranibizumab, as well as triamcinolone, appears to reduce the risk of worsening diabetic retinopathy [35].

### 3.3. Bevacizumab

Intravitreal bevacizumab (IVB) has been widely used off-label for the treatment of AMD, especially because of its significantly lower cost compared to ranibizumab, in addition to positive clinical effects demonstrated in early studies [36]. The widespread use of IVB for the management of DME led to the need of a formal evaluation of its safety and efficacy [37–39].

DRCR.net conducted a phase II exploratory trial including 121 eyes with DME over a 12-week period to assess the short-term effect of IVB [39]. The eyes were randomized into five groups: (I) focal laser, (II) two intravitreal injections of 1.25 mg of bevacizumab at 0 and 6 weeks, (III) two intravitreal injections of 2.5 mg of bevacizumab at 0 and 6 weeks, (IV) 1.25 mg of bevacizumab at week 0 followed by a sham injection at week 6, and (V) 1.25 mg of bevacizumab at 0 and 6 weeks plus focal laser at 3 weeks. Eyes assigned to groups II and III had a significant BCVA improvement over the laser-only group I, and this difference was seen throughout the 12 weeks. These two groups also had a greater improvement in central subfield thickness (CST) at the 3-week visit. No differences were seen between groups 1.25 mg and 2.5 mg bevacizumab. The single injection group was not superior to the laser group. Bevacizumab plus laser showed results comparable to laser-only treatment. This study suggested that bevacizumab was an effective drug for treating DME as a primary treatment and also for refractory eyes, since 69% of included eyes were refractory to previous treatment. However, eyes that received primary treatment had greater improvement than the refractory ones (*P* = 0.04). No safety concerns were detected in 24 weeks. Similar outcomes showing no difference between 1.25 mg and 2.5 mg of bevacizumab have been previously reported in other studies [40, 41].

A randomized clinical trial compared IVB injection alone or in combination with intravitreal triamcinolone acetonide (IVT) versus macular laser photocoagulation as a primary treatment for DME. A total of 150 eyes were randomly assigned to the following groups: (I) 1.25 mg IVB, (II) IVB/IVT, with 1.25 mg IVB and 2 mg IVT, and (III) macular laser. The IVB group showed significant superiority in visual acuity improvement after six months, but this was not sustained after 24 months. The mean BCVA was significantly better in the IVB-only group compared to baseline, after 24 weeks [7, 42].

The study conducted by the Pan-American Collaborative Retina Study Group (PACORES) examined IVB as the primary treatment for diffuse DME at 11 centers in 8 countries [38]. This retrospective, multicenter, interventional, comparative case series reviewed clinical data of 139 eyes with diffuse DME treated with at least 1 off-label intravitreal injection of either 1.25 or 2.5 mg of bevacizumab. The dose received at baseline was the same dose delivered throughout the study. Follow-up considered BCVA measurement with ETDRS charts and OCT at baseline and 1, 3, 6, 12, and 24 months after the initial injection. The reinjection criterion was recurrence of diffuse DME [43].

No significant differences between the 1.25 mg and 2.5 mg dose groups were detected. Mean BCVA and central macular thickness (CMT) improved at 1 month after the first IVB and such significant outcomes were sustainable all along the 24 months; when the results demonstrated that 72 (51.8%) eyes improved by 2 or more ETDRS lines, 62 (44.6%) eyes remained stable, and 5 (3.6%) eyes decreased by 2 or more ETDRS lines of BCVA. At 24 months, OCT analysis showed that CMT decreased from 446.4 ± 154.4 to 279.7 ± 80 *μ*m. The mean number of injections per eye was 5.8 (range of 1–15 injections) at a mean interval of 12.2 ± 10.4 weeks [43].

The bevacizumab or laser therapy (BOLT) study is a prospective, randomized, blinded, single-center study that compared IVB to macular laser photocoagulation in patients with persistent DME after at least one macular laser treatment [44]. Eighty eyes were randomized into either the bevacizumab group, receiving injections every 6 weeks, with a minimum of 3 and a maximum of 9 injections, or the laser group, receiving treatment every 4 months, with a minimum of 1 and a maximum of 4 treatments. Mean BCVA after 1 year increased in the bevacizumab group and declined in the laser group. The CMT results were superior in the bevacizumab group as well. The mean number of interventions was 9 injections and 3 laser treatments during the first year.

The 2-year outcome report from the BOLT study was published in 2012 and presented similar results to those obtained in the first year report [45]. The mean BCVA was 20/50 in the group treated with bevacizumab and 20/80 in the laser group (*P* = 0.005), with a mean gain of 8.6 letters for bevacizumab versus a mean gain of 0.5 letters for the laser group. Regarding improvement of 15 letters or more, 32% of the eyes treated with bevacizumab achieved this target versus 4% for the laser-treated eyes (*P* = 0.004). On the other hand, the proportion of subjects that lost fewer than 15 letters in the laser group was 86% versus 100% for the bevacizumab group (*P* = 0.03). CMT decreased significantly in both groups at 2-year follow-up and the mean number of treatments was 13 injections and 4 macular laser interventions. These outcomes provided by the BOLT study support the longer term use of IVB for the treatment of DME.

### 3.4. Aflibercept

Encouraged by positive results from a phase I study [46], a phase II, multicenter, randomized clinical trial was conducted to investigate different dosing regimens of intravitreal VEGF Trap-Eye for the treatment of DME compared to standard macular laser [24]. The DA VINCI (DME A and VEGF Trap-Eye: Investigation of Clinical Impact) study enrolled 221 subjects with center-involved DME and BCVA between 20/40 and 20/320 randomized into 5 groups: 0.5 mg VEGF Trap-Eye every 4 weeks (0.5q4), 2.0 mg VEGF Trap Eye every 4 weeks (2q4), 2.0 mg VEGF Trap Eye monthly for 3 months and then every 8 weeks (2q8), 2.0 mg VEGF Trap Eye monthly for 3 months and then as needed (2 PRN), and macular laser treatment. All VEGF Trap Eye groups received sham laser and all laser patients received sham injection. The primary endpoint was mean change in BCVA. The change from baseline in central retinal thickness and proportion of patients gaining at least 15 letters at week 24 were among secondary outcomes [24].

Improvement in mean change of BCVA was observed ranging from 8.5 to 11.4 letters in groups receiving aflibercept versus 2.5 letters in the laser group, at week 24. Central retinal thickness significantly decreased more in the groups treated with VEGF Trap-Eye compared to the laser group. No significant differences were seen between the aflibercept groups, supporting a treatment regimen of every 8 weeks instead of every 4 weeks [24]. At 52 weeks, the change in mean BCVA ranged from 9.7 to 13.1 letters in the aflibercept groups versus a loss of 1.3 letters in the laser group [47].

Two phase III trials are ongoing and have recently divulged early outcomes. VIVID-DME (VEGF Trap-Eye in Vision Impairment due to DME), in Europe, Japan, and Australia, and VISTA-DME (Study of Intravitreal Administration of VEGF Trap-Eye in Patients with Diabetic Macular Edema), in USA, are randomized, double-masked, active controlled trials that investigate the efficacy and safety of repeated doses of intravitreal VEGF Trap-Eye in subjects with DME [48]. The trials are both sponsored by Bayer (Bayer AG, Leverkusen, Germany) and Regeneron Pharmaceuticals (Regeneron Pharmaceuticals, Inc., Tarrytown, USA) and may support the FDA approval for the use of aflibercept in DME [49, 50].

VIVID-DME enrolled 404 patients and VISTA-DME 461, randomized (1 : 1 : 1) to receive intravitreally either 2.0 mg aflibercept every 4 weeks (2q4) or 2.0 mg aflibercept every 8 weeks after 5 initial monthly doses (2q8) or laser photocoagulation. Primary endpoint was mean change in BCVA at week 52. The patients were scheduled for continued treatment for 3 years [48]. After the first year, the primary results showed the superiority of aflibercept groups over the laser treatment group. Mean change in BCVA in the VIVID-DME study was plus 10.7 letters in the 2q8 group and plus 10.5 in the 2q4 versus plus 1.2 in the laser group. The VISTA-DME showed a similar mean change in BCVA of plus 10.7 letters in the 2q8, plus 12.5 in the 2q4, and plus 0.2 in the laser group. The mean change in central retinal thickness, proportion of patients gaining at least 15 letters, and improvement of Diabetic Retinopathy Severity Score (DRSS), all secondary outcomes, showed a significant superiority of aflibercept over laser treatment. On average, the aflibercept 2q8 group performed similarly as the aflibercept 2q4 group. No systemic safety signal was detected in either aflibercept treatment group through week 52 [48].

## 4. Further Study and Concerns

The efficacy of intravitreal anti-VEGF for the treatment of DME has recently been proved by various studies. Safety issues concerning intravitreal injection of anti-VEGF are well known from AMD studies, although none of those trials or DME trials had enough power to detect significant differences between the study groups regarding adverse events. Serious ocular adverse events are of low frequency and include endophthalmitis, uveitis, and retinal detachment; likewise the risk of occurrence does not seem to be greater in patients with DME than in AMD. Serious systemic adverse events could be death, myocardial infarction, and stroke. Most safety studies, however, have failed to identify issues regarding such systemic events related to intravitreal anti-VEGF injection [32, 36, 47, 48].

The small number of relevant trials and variation in their characteristics limit comparisons between different anti-VEGF drugs. A relevant ongoing study conducted by the DRCR.net, the protocol T, proposes as its primary objective to compare the efficacy and safety of intravitreal aflibercept, bevacizumab, and ranibizumab when used to treat central-involved DME. To date, 660 subjects with DME and BCVA between 20/32 and 20/320 were enrolled and will be randomized to receive either 1.25 mg bevacizumab, or 0.3 mg ranibizumab, or 2.0 mg aflibercept [51]. Considering that ranibizumab is the only approved anti-VEGF for DME, the markedly lower cost of bevacizumab, and the potential of aflibercept to decrease treatment burden and associated cost, the outcomes from this study should have an extensive impact on clinical practice regarding the management of DME.

## 5. Conclusions

Diabetic macular edema is an important cause of vision impairment and macular photocoagulation has been the standard treatment for this condition. Recent studies have presented significantly positive visual and anatomical results regarding the use of anti-VEGF for the treatment of DME, both as primary intervention and in refractory cases. Although the protocols are all consistently different, undoubtedly anti-VEGF therapy has assumed an important role in the management of DME, either as a first choice or as adjuvant to photocoagulation.

This review was conducted to better understand the impact of the outcomes of recent trials on clinical practice. Further studies are necessary, especially to investigate long-term efficacy and safety, to compare drugs, and establish guidelines. However, confronted with a diagnosis of center-involved diabetic macular edema, it has become mandatory to consider treatment with intravitreal anti-VEGF injections.

## Figures and Tables

**Table 1:** Anti-VEGF agents.

Anti-VEGF agents	Mechanism of action	Molecular weight	FDA approval
Pegaptanib sodium (Macugen)	Selective VEGF antagonist (165 isoform).	50 kDa	AMD (2004)

Ranibizumab (Lucentis)	Recombinant humanized IgG1 kappa antibody fragment. Inhibits all isoforms of human VEGF-A.	48 kDa	AMD (2006) RVO edema (2010) DME (2012)

Bevacizumab (Avastin)	Full-size, humanized, recombinant monoclonal IgG antibody. Inhibits all isoforms of human VEGF-A.	149 kDa	Off-label use in ophthalmology

Aflibercept (Eylea)	Fully human recombinant fusion protein. Inhibits all isoforms of human VEGF-A and B as well as binds placental growth factors 1 and 2.	115 kDa	AMD (2011) RVO edema (2012)

RVO: retinal vein occlusion.
